# Supplementary material for: Transcriptomic Insights into Mechanisms of Early Seed Maturation in the Garden Pea (Pisum sativum L.)
Source: Cells. 2020 Mar 23;9(3):779. doi: 10.3390/cells9030779 (PMC7140803; doi:10.3390/cells9030779)

Sprint−2,20(1)

Global Summary

%DE = 0.25

genes with fdr < 0.2 = 530 (230 + / 300 −)

genes with fdr < 0.1 = 459 (210 + / 249 −)

genes with fdr < 0.05 = 422 (194 + / 228 −)

genes with fdr < 0.01 = 365 (169 + / 196 −)

genes in genesets = 0

<FC> = 0

<t−score> = 0

<p−value> = 0

<fdr> = 0.75

Portrait

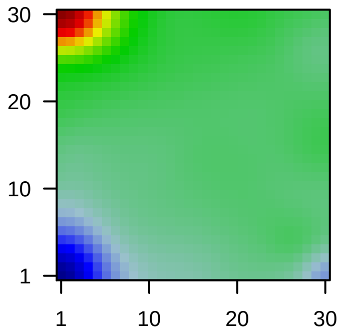

Top 100 DE genes

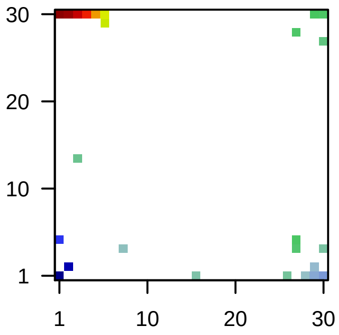

Global Genelist

Rank	ID	log(FC)	p−value	fdr	Metagene	Description
1	TRINITY_DN1	0.61	2e−16	2e−15	30 x 1	
2	TRINITY_DN2	−0.57	2e−16	2e−15	1 x 1	
3	TRINITY_DN9	−0.7	2e−16	2e−15	30 x 30	
4	TRINITY_DN7	0.97	2e−16	2e−15	30 x 1	
5	TRINITY_DN7	1.64	2e−16	2e−15	30 x 30	
6	TRINITY_DN5	0.48	2e−16	2e−15	30 x 30	
7	TRINITY_DN5	−0.83	2e−16	2e−15	1 x 1	
8	TRINITY_DN5	0.8	2e−16	2e−15	1 x 30	
9	TRINITY_DN7	−1.2	2e−16	2e−15	1 x 1	
10	TRINITY_DN5	−0.4	2e−16	2e−15	30 x 4	
11	TRINITY_DN4	−2.43	2e−16	2e−15	1 x 1	
12	TRINITY_DN9	1.51	2e−16	2e−15	4 x 30	
13	TRINITY_DN9	−0.79	2e−16	2e−15	30 x 1	
14	TRINITY_DN9	−0.49	2e−16	2e−15	30 x 30	
15	TRINITY_DN9	−0.84	2e−16	2e−15	30 x 30	
16	TRINITY_DN9	1.42	2e−16	2e−15	27 x 4	
17	TRINITY_DN2	2	2e−16	2e−15	1 x 30	
18	TRINITY_DN2	−0.64	2e−16	2e−15	1 x 1	
19	TRINITY_DN2	−0.54	2e−16	2e−15	30 x 1	
20	TRINITY_DN2	1.17	2e−16	2e−15	30 x 30	

p−values

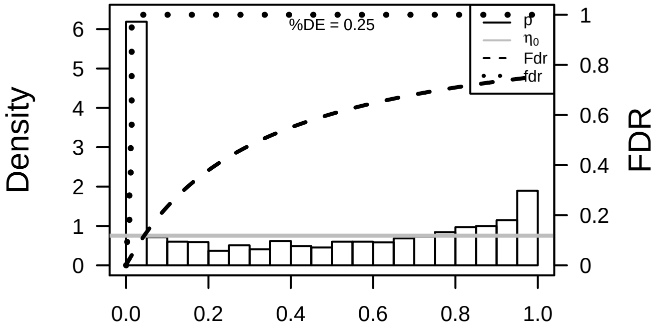

Supplement: Supplementary file 1 [file cells-09-00779-s001.zip › Supplementary materials/FileS4.zip!/FileS3/TFs - Results/Summary Sheets - Samples/Sprint.2.20.1..pdf]
